# The price of safety and convenience: Urban shoppers’ willingness to pay for hygienic market stalls and minimal processing of leafy vegetables in Kenya

**DOI:** 10.1371/journal.pone.0340495

**Published:** 2026-03-10

**Authors:** Mercy Mwambi, Paul Opiyo, Augustine Wafula, Pepijn Schreinemachers, Ralph Roothaert, Julia de Bruyn

**Affiliations:** 1 World Vegetable Center, Arusha, Tanzania; 2 Jaramogi Oginga Odinga University of Science and Technology, Kisumu, Kenya; 3 World Vegetable Center, Nairobi, Kenya; 4 World Vegetable Center, Bangkok, Thailand; 5 Mukinduri Consulting Africa, Nairobi, Kenya; 6 University of Reading, Reading, United Kingdom; University of Georgia, UNITED STATES OF AMERICA

## Abstract

The safety of fresh food is a serious concern across sub-Saharan Africa. Stronger incentives are needed to stimulate market retailers to adopt more hygienic practices and to drive improvements in fresh produce market infrastructure. A price premium for safe produce could incentivize retailers to do this, but it is unknown if shoppers would accept a higher price. The objective of this study was to evaluate the demand for safer vegetables among urban consumers in Africa. The Becker–DeGroot–Marschak (BDM) method was used to conduct a non-hypothetical experimental auction with 417 randomly selected shoppers in five fresh produce markets in Kisumu, Kenya. Shoppers chose between two bundles of mixed African leafy vegetables: (1) whole, unpackaged, and (2) minimally processed vegetables – washed, plucked, and packaged; and two food safety conditions: (3) conventional handling practices in a traditional stall, and (4) hygienic handling in an improved stall based on government guidelines. The results show that shoppers are willing to pay about 24% more for whole vegetables from clean stalls and about 26% more for minimally processed vegetables. Plucked vegetables pose a higher food safety risk than whole vegetables, but wealthier, time-constrained shoppers opt for them for convenience. These findings indicate that urban shoppers in Kenya attach value to the safety of fresh produce. Retailers can command higher prices by enhancing stall hygiene, but external support will be needed to upgrade retail market infrastructure.

## 1. Introduction

Urban consumers across Africa are increasingly interested in buying fresh, nutritious, and safe food. This demand is driven by urbanization, shifting dietary preferences, and increased awareness of foodborne health risks. Vegetables, especially African leafy vegetables (ALVs), are crucial in providing essential micro- and macronutrients for human health [1]. They are also culturally accepted, suitable for local production environments, and sometimes have other health and medicinal properties [2,3]. Despite their advantages, ALV consumption remains low. The overall vegetable consumption of the Kenyan population is also low, at around 120 g per adult per day, well below the 240 g/day generally recommended by WHO and FAO [4].

Safety perceptions play a key role in people’s decisions to consume vegetables. Urban consumers are increasingly aware of risks associated with chemical residues and foodborne illnesses caused by human pathogens transmitted through contaminated food [5]. Cooking reduces pathogen risks, but ALVs are sometimes not cooked at sufficient heat [6] and pathogens on fresh vegetables cross-contaminate other foods in the kitchen [7]. Microbial contamination can occur at multiple stages along the value chain, from the farm through the use of contaminated irrigation water [8] to other supply chain nodes through poor postharvest hygiene and handling practices [9]. At the market level, several practices are of concern: vendors often sprinkle leafy vegetables with water to improve their appearance and reduce wilting, but this can introduce or spread harmful microbes. Market stalls and storage facilities made of materials such as wood and plastic are difficult to sanitize, undermining efforts to maintain food safety by minimizing microbial contamination [10] while open-air trading further exposes fresh produce to environmental contaminants [11]. Several studies have shown an elevated risk of microbial contamination of vegetables sold in informal markets with poor hygiene [11–14].

Another constraint on urban consumption is the labor-intensive preparation required for ALVs. Preparation often involves several rounds of washing, as these vegetables are usually sold in uprooted form [2], and removing the leaves from the stalks (commonly referred to as ‘plucking’), chopping, and draining [15]. These steps are time-consuming, especially for urban dwellers who commute long distances and have little time to cook [16]. Plucked or chopped vegetables offer a time-saving solution, making convenience an important attribute in fast-paced urban environments. Fresh produce market vendors sometimes offer washing, plucking and chopping services, which, while convenient, expose more of the vegetable’s surface area to bacterial contamination [17]. A study in Nairobi, Kenya, found that water used to wash vegetables in markets was contaminated with microbial pathogens, including *Salmonella* spp. [18]. Reusing the same water multiple times to wash vegetables creates another food safety hazard.

In many African cities, vegetables are sold through informal market chains where food safety standards and regulations are nearly non-existent or poorly enforced, and where there is inadequate infrastructure, such as potable water, waste disposal facilities, and drainage [19,20]. While food safety standards may mandate that markets have clean on-site toilets and handwashing facilities, recent studies show that these are often absent [10,21]. Despite these weaknesses, the informal chain continues to provide access to diverse, nutritious, and affordable fresh foods for most urban consumers [22]. Given the importance of informal supply chains and complex hygiene conditions in fresh produce markets, it is crucial to determine whether and how much consumers are willing to pay a premium for products with bundled attributes of safety and convenience.

The objective of this study is to evaluate the demand for safe vegetables among urban consumers in Africa. Kisumu, Kenya, presents a suitable case due to its rapid urban growth, evolving food systems, and reliance on informal supply chains [23]. ALVs offer an interesting case study crop because demand for ALVs is increasing, and they are popular among low-income consumers [24]. The study examines two specific attributes: 1) **hygiene**, signaled through an improved stall with a handwashing set, clean protective clothing, and elevated storage; and 2) **convenience**, represented by plucked, washed and packaged vegetables that save consumers’ time in food preparation.

Previous studies have shown evidence of consumers’ willingness to pay for food safety attributes, but the focus has been on products free of pesticides, as signaled through labeling. Urban consumers were shown to be willing to pay more for organic-labeled tomatoes than traditional ones in central Tanzania [25] and 50% more for pesticide-free vegetables in urban and peri-urban Benin and Ghana [26]. A study in Vietnam conducted a hypothetical valuation and found that consumers were willing to pay 60% more for Chinese mustard free of pesticide residues and 19% more for convenience [27].

Vendors could be incentivized to maintain food safety if consumers are willing to pay more for hygiene-related measures. Without strict monitoring and control standards, monetary incentives could provide a pathway to food safety in well-functioning markets [19]. If observable food safety characteristics are demonstrated to be positively associated with willingness to pay, then producers and retailers may be willing to invest in infrastructure upgrades and adopt improved food safety practices. However, caution is needed with price-based food safety mechanisms because they could prevent low-income consumers from accessing the product.

## 2. Materials and methods

### 2.1. Methodological approach

The study is grounded in Lancaster’s theory of consumer demand, which holds that a good in itself does not provide utility to the shopper but rather possesses attributes that satisfy needs and wants [28]. Following this theory, this study treats vegetables as bundles of food safety and convenience attributes that shape consumers’ purchasing behavior. A rational shopper will choose the bundle of attributes that maximizes utility subject to factors such as income, household size, and other socioeconomic or cognitive factors [16]. The economic theory of opportunity cost implies that rational consumers weigh the benefits of safety and convenience against the additional costs associated with them. For instance, consumers may be interested in plucked and washed ALVs to reduce preparation time, but the extra cost may be a barrier. Alternatively, consumers may prioritize safety over convenience, paying more for whole vegetables from safe environments even if they require more time to prepare at home.

The Becker-DeGroot-Marschak (BDM) approach was used to elicit participants’ willingness-to-pay for the above attributes. BDM is widely used to assess willingness to pay for safe and quality food products in low- and middle-income countries [25,29] because it allows individual bidding as opposed to group bidding. The willingness to pay elicitation mechanism can typically be done in two ways: a) full bidding, in which the subject bids for an existing product and a new, improved product, and the difference in the bids is the measured premium; b) endow and upgrade, in which the subject is endowed with the existing product and bids to exchange it for the new and improved product. While each method has its advantages, the full bidding approach was selected as it avoids the reverse endowment effect that happens when using endow and upgrade [30,31].

### 2.2. Study site and background

The study was implemented in Kisumu City, the third-largest city in Kenya after the capital Nairobi and the coastal city of Mombasa. Kisumu has one of Kenya’s highest poverty levels, estimated at 48%, with over 60% of the city population living in peri-urban settings.

The selection of vegetables was guided by a ranking based on competitiveness (high market demand, growth potential, etc.), impact potential (poverty reduction, employment creation, etc.), food security (e.g., availability and access to food, lower food prices) and cross-cutting issues (e.g., gender inclusivity) [32]. The ranking, conducted by local stakeholders, identified ALVs as the most important in Kisumu.

African vegetables are commonly produced, marketed and consumed in Kisumu. A recent study estimates that 2 tons of African vegetables are traded daily in Kisumu city markets, generating USD 21,663 (KES 2.8 million) in weekly revenues for traders [32]. Nearly all African vegetables sold in Kisumu are sourced from sub-counties in Kisumu and nearby counties like Vihiga and Kisii. Most vegetables are sold in fresh produce markets.

Market vendors in Kisumu display a range of different ALVs tied in small bunches, with consumers commonly purchasing multiple bunches, according to their preferred combination. Alternatively, the vendor may select a variety of vegetable bunches on their behalf. A common combination consists of amaranth (*Amaranthus viridi*s) (40%), black nightshade (*Solanum nigrum)* (40%), and spider plant (*Cleome gynandra L)* (20%). Therefore, to mimic the real market situation, the experimental product consisted of a mix of these three vegetables. For this study, each product bundle weighed 500 grams.

The vegetables used in this experiment were purchased from a group of farmers who follow agroecological production practices and were trained by the project for several years. The group delivered fresh vegetables for the experiment every morning, and the products were stored in a cooler to maintain quality. Netting bags, a common packaging material in Kenya, were used to package the products in 500g units.

**Fig 1:**
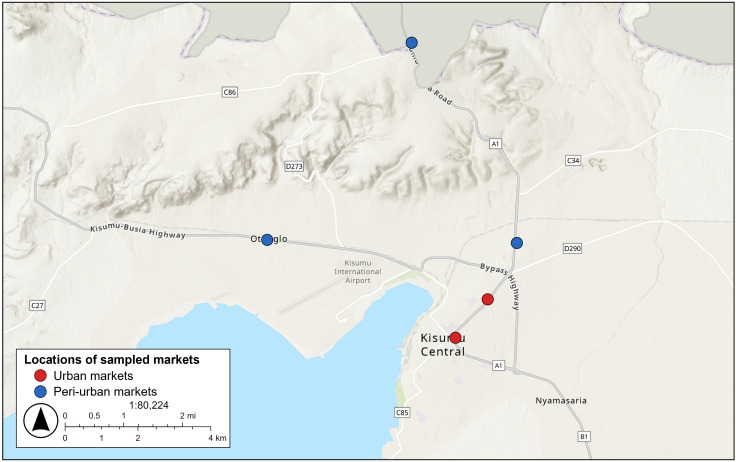
Basemap: Esri World Topographic Map (© Esri and its data providers).

Out of 21 retail markets in Kisumu, two large markets were selected in the city center (Jubilee and Kibuye markets), and three smaller markets were selected from peri-urban areas (Mamboleo to the east, Kiboswa to the north, and Otonglo to the west) (Fig 1). The choice of markets ensured a diverse range of shoppers in high-end and low-income areas of Kisumu.

### 2.3. Product attributes tested

**Fig 2:**
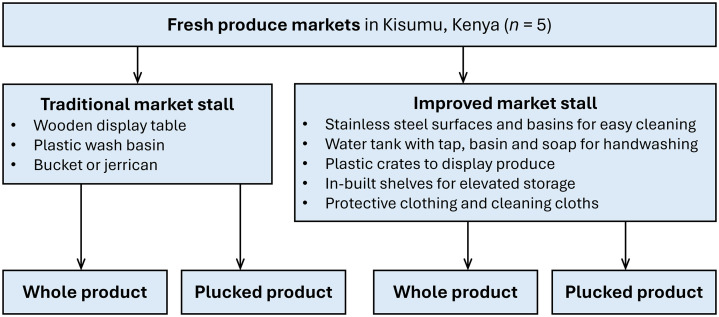
Experiment stall and product arrangement.

The experiment was designed to value two product attributes: convenience and hygiene. Each attribute was presented as two choices. Hygiene involved a choice between traditional and improved market stalls (Fig 2; S1 Fig). Convenience involved a choice between whole and minimally processed vegetables, basically, washed and plucked. Each stall type offered both types of vegetables.

The stalls were located at regular fresh produce markets. The traditional market stall consisted of a wooden structure, a wooden display table, and a basic set of equipment (a plastic wash basin, a 20-litre jerrican or bucket, and knives). The improved stall was designed and implemented to comply with food safety measures covering personal hygiene, water, and equipment, as guided by the local government food safety expert and as based on the Food, Drugs and Chemical Substances Act of Kenya [33]. It had a steel display surface and stainless-steel basins and knives—all of which could be easily cleaned. It also included a handwashing and stall hygiene set, consisting of a 50-liter water tank with a tap, table wipes, and soap. Plastic crates were provided for displaying leafy vegetables, while in-built storage allowed excess produce to be stored above ground level. Protective clothing (a clean apron and hair cover) was provided to vendors at the improved stall during the experiment. Vendors and shoppers were motivated to clean their hands before touching the produce.

Each respondent was informed about the different product attributes and about the improved stall. Two enumerators were assigned to share information. For food product attributes, enumerators explained that one product is whole and the other is plucked. The enumerators provided respondents with information about the improvements made to the upgraded stall. This included the installation of a handwashing station, an easy-to-clean display table and wash basin, raised storage, and proper attire for the vendor, and the importance of these improvements in reducing food contamination.

### 2.4. Sampling and data collection

Power calculations were performed in Optimal Design software, assuming a multi-site, personalized, randomized controlled design. To detect an effect size of 0.40 with a power of 0.80, a sample of 300 participants was required. The study targeted 400 participants to ensure a sufficiently large sample, but the eventual sample had 417 participants. Kibuye and Jubilee markets had larger samples reflecting their larger size (Table 1).

**Table 1:** Sample size distribution over five Kisumu retail markets.

Market	Location	Targeted respondents	Actual respondents
Kibuye	Urban	170	177
Jubilee	Urban	140	123
Mamboleo	Peri-urban	30	42
Kiboswa	Peri-urban	30	39
Otonglo	Peri-urban	30	36
**Total**		**400**	**417**

A voucher worth USD 0.92 (KES 120), which is 20% more than the highest price of a mixed bunch of ALVs in the retail outlets, was given to each participant before the experiment as a token of appreciation for participating in the interview. Participants were informed that they could redeem the voucher to buy vegetables in case they won the bid. The participants were asked two screening questions: whether they were the person of their household primarily responsible for vegetable purchases, and whether they had consumed ALVs in the past month. Those who answered “yes” to both questions were interviewed. Participants were briefed on the experiment and the rules. Questions were asked to evaluate participants’ understanding of the experiment.

Once ascertained that participants understood the task, the researcher asked the participants to observe the two stalls and the products displayed in each. The researcher described the product and the hygiene practices of the improved stall, then the participants were invited to inspect the product and hygiene measures. Participants submitted price bids for all products and then drew a random number from a bowl containing product numbers (1, 2), which was used to select a binding product. The participants then drew a random price from a uniform distribution of prices of 500g mixed vegetable bundles out of the price pool. These prices had been obtained from different outlets in the study area. Participants who submitted a bid at or above the drawn price purchased the selected vegetable product at the drawn price and redeemed the balance on their voucher. Those who submitted a bid price lower than the randomly drawn price did not buy the selected product but redeemed their voucher.

After the experiment was completed, brief interviews using a structured questionnaire were administered to collect data on various aspects, such as awareness of food safety, perceptions, and economic characteristics. The experiment was done on weekdays and weekends and at different times of the day to cover a range of different shoppers. The products were presented alternately to participants to avoid potential bias.

Several sources of bias can influence willingness-to-pay bids in experimental auctions if not properly addressed in the experiment design. Order effects bias can occur from the sequence in which items are presented to respondents. To prevent this, the products and stalls were presented alternately to participants. Alternating the sequence also helps mitigate attenuation bias by reducing the likelihood that safer products always follow traditional products, or vice versa.

‘House money’ effects can happen when participants spend some of the money they unexpectedly received in the experiment because they don’t see it as their own, or because they want to reciprocate to the experimenter [34]. To address this, shoppers were provided with a voucher instead of cash at the start of the experiment and reminded that they could use it as they saw fit.

Another source of bias is when participants misunderstand the structure of the BDM experiment, which is known as the ‘game form recognition’ problem [35]. Participants were asked two basic questions about the experiment to assess their understanding. If the participant failed to answer either question correctly, the enumerator explained the bidding exercise again. Enumerators only proceeded when both questions were answered correctly.

Participants may respond differently in an experiment than in a real situation. This was addressed by introducing ‘cheap talk’, which is communication that has no real-world consequences or impact on the outcome of a game, and by explicitly explaining hypothetical bias to individuals before bidding [36]. The cheap talk contained information such as “We encourage you to provide your true bid for this product. Your bid should reflect the amount you are truly willing to pay if you had the opportunity to purchase this product on display shelves in a similar market tomorrow”. This was meant to encourage truthful bidding.

The interviews took place from September 6–19, 2024. An information sheet was read to the respondent to explain the study’s objective, the methods used, and the risks and benefits associated with participation. Confidentiality and data usage details were also included. Informed consent was sought to indicate that the participants understood the study as described in the information sheet. Consent was recorded as yes or no in the digital data collection tool, and participants who answered yes were allowed to participate. This study was approved by the Institutional Biosafety and Research Ethics Committee (IBREC) of the World Vegetable Center (Registration no. 2024−013). Information regarding ethical, cultural, and scientific considerations specific to inclusivity in global research is included in the Supporting Information (S1 Checklist).

### 2.5. Data analysis

The relationship between willingness to pay for different attributes and socioeconomic characteristics can be specified as:


$${WTP}_{ij}=\;\alpha +{\beta Z}_{ij}+{\gamma Z}_{i}+\;\varepsilon_{ij},$$ (1)

where ${WTP}_{ij}$ represents shopper *i*’s willingness to pay for vegetable product *j* and refers to the bids; ${\beta Z}_{ij}$ is a vector of shoppers’ demographics and $\varepsilon_{ij}$ is the error term. Separate models were run for each product in the improved stall to assess the determinants of willingness to pay.

The questionnaire survey captured diverse aspects of consumer behavior and perceptions. Information on demographic characteristics such as gender, age, level of education, marital status, household size, having a child under 5 years, having a vegetarian household member, and income was collected, as previous studies found these to influence consumer behavior regarding food safety [37,38].

Summary statistics in Table 2 reveal that 62% of respondents were married, 83% were female, and 57% were under 35 years old. About a third of the respondents had completed education beyond secondary school, although 90% could read and understand English and Swahili. Most respondents belonged to the high-income bracket based on their residence location. The average household had five members, and 17% employed a domestic helper. Most households did not have a vegetarian member (62%). Respondents reported that at least one household member experienced symptoms of foodborne illness in the past six months, with the most common being stomachache (57%), diarrhea (42%), nausea (29%), and vomiting (21%). There was a strong preference for buying from improved stalls when available (93%), but only 23% preferred purchasing plucked vegetables.

**Table 2:** List and nature of variables used in the analysis.

Variable description	Unit	Mean (SD)
Sex of the respondent	1 = female	0.83
Age of respondent	1=> 35 years; 0 = 35 years and below	43.0
Education level	1 = post-secondary, 0 = secondary and below	0.35
Marital status	1 = married	0.62
Household size	Number of persons	4.92 (2.72)
Household has a vegetarian member	1 = yes	0.38
Household has a domestic helper	1 = yes	0.17
Household has children under 5 years	1 = yes	0.56
Can read English	1 = yes	0.90
Can read Swahili	1 = yes	0.95
Respondent lives in a high-income residential area	1 = high income, 0 = otherwise	0.71
Extent to which respondent is concerned about eating contaminated vegetables	1 = never, 2 = sometimes, 3 = always	2.73
Food safety knowledge (+)	1 = yes, 2 = no, 3 = don’t know	0.87
Food safety knowledge (-)	1 = yes, 2 = no, 3 = don’t know	0.39
Food safety perception (+)	1 = strongly disagree, …, 5 = strongly agree	4.67
Food safety perception (-)	1 = strongly disagree, …, 5 = strongly agree	1.65
Importance of product quality attributes	1 = very unimportant, 5 = very important	3.87
Importance of socioeconomic attributes	1 = very unimportant, 5 = very important	3.36
Would prefer buying from an improved stall in the future	1 = yes	0.93
Bought plucked vegetables in the past month	1 = yes	0.23
Symptoms consistent with foodborne illness experienced by any household member in the past six months:		
- Stomachache	1 = yes	0.57
- Diarrhea	1 = yes	0.42
- Nausea	1 = yes	0.29
- Vomiting	1 = yes	0.21

Food safety knowledge was assessed through a series of 11 statements, to which consumers indicated whether each was true. Three answers were possible: “yes”, “no”, and “don’t know”. These statements, developed by the researchers based on market observations and consultation with local experts, specifically addressed respondents’ knowledge of cross-contamination and hygienic practices. Examples include ‘*hand hygiene can reduce vegetable contamination*’, ‘*pathogenic bacteria in vegetables are killed off completely if I cook my vegetables*’, ‘*using clean water to wash vegetables makes vegetables safe*’, etc. (S1 Table). Each correct answer scored 1 point, giving a food safety knowledge score of 0–11.

In addition, consumers were asked about their frequency of concern about eating contaminated vegetables (“never”, “sometimes” or “always”) and whether anyone in their household had experienced symptoms consistent with food-borne disease in the past six months (yes/no). Finally, participants were asked to indicate the extent to which they agreed or disagreed with statements related to food safety, using a five-point Likert scale, where 1 is completely disagree and 5 is completely agree. Questions included ‘*frequent cleaning of the display tables maintains the safety of vegetables*’ and ‘*placing vegetables on a sack or mat on the ground reduces safety*’ (S2 Table). In total, nine statements were asked.

Shoppers were asked how important product quality attributes are to them, and they responded on a 5-point Likert scale, where 1 = very unimportant and 5 = very important. Freshness, nutrient value, and taste were among the quality attributes, while the size of the bunch, the price of vegetables, and stall and market hygiene were among the market attributes (S3 Table). Principal component analysis (PCA) was used to generate composite indices and consolidate responses to Likert-scale questions on food safety knowledge, perceptions of product quality attributes, and market attributes. The indices were included in the model.

Price data for African vegetables were collected from nine retail stores in Kisumu’s urban and peri-urban areas. At each outlet, the price was recorded per bunch of amaranth, black nightshade, and spider plant, and the weight of each bunch was taken using a weighing scale to assist in computing a standard measure of price per 500g of vegetables.

## 3. Results

### 3.1. Vegetable purchasing

On average, shoppers bought four bunches of amaranth in their last purchase, while for African nightshade and spider plant, they bought an average of five bunches (Table 3). The average purchase price reported was approximately USD 0.08 per bunch across all vegetable types. The average weight per bunch was 151g for amaranth, 177g for nightshade, and 154g for spider plant. Participant-reported prices ranged from USD 0.23 to 0.26 for 500 g of leafy vegetables.

**Table 3:** Vegetable purchasing patterns of sampled shoppers, n = 417.

Purchasing Patterns	Mean	Standard deviation	Maximum	Minimum
**(a) Quantity of most recent purchase (bunches);**				
- Amaranth	4.21	4.24	50	0
- Nightshade	5.62	7.23	120	0
- Spider plant	4.98	6.12	80	0
**(b) Price of last purchase (USD/bunch):**				
- Amaranth	0.08	0.03	0.38	0.02
- Nightshade	0.08	0.05	0.77	0.03
- Spider plant	0.08	0.05	0.77	0.03
**(c) Price of last purchase (USD/500g equivalent):**				
- Amaranth	0.26	0.10	1.26	0.07
- Nightshade	0.23	0.14	2.18	0.08
- Spider plant	0.26	0.16	2.50	0.10

Note: Average weight/bunch of amaranth = 151 g; nightshade = 177 g; spider plant = 154 g as measured from retail vendor outlets

### 3.2. Bid distribution

Table 4 shows the distribution of bids for the different products. The results show that plucked vegetables attracted a higher mean price than whole vegetables across both stall types. It also shows that the improved stall elicited higher mean price bids for all products than the traditional stall, reflecting a preference for enhanced hygiene. Shoppers were willing to pay 23.5% more for whole vegetables in the improved stall compared to those in the traditional stall (p < 0.001) and 25.7% more for plucked vegetables in the improved stall compared to those in the traditional stall (p < 0.001). There were differences in willingness to pay for products within stalls. Shoppers offered to pay 2.9% and 4.8% more for the plucked vegetables compared to whole ones in the traditional and improved stalls, respectively (p = 0.010).

**Table 4:** Mean price elicited by shoppers for the different products, n = 417.

Products	Mean (USD)	Standard deviation	Maximum (USD)	Minimum (USD)
**Traditional stall:**				
- Whole vegetables	0.34	0.10	0.62	0.15
- Plucked vegetables	0.35	0.13	0.62	0.15
**Improved stall:**				
- Whole vegetables	0.42	0.15	0.77	0.23
- Plucked vegetables	0.44	0.19	0.85	0.15
**Comparisons**	**Mean difference (USD)**	**Mean difference (%)**	**p-value**	**Significance**
**Traditional vs. improved stall:**				
- Whole vegetables	0.08	23.5	<0.001	***
- Plucked vegetables	0.08	25.7	<0.001	***
**Whole vs. plucked vegetables:**				
- Traditional stall	0.01	2.9	0.010	**
- Improved stall	0.02	4.8	0.010	**

Note: All prices in US dollar per 500g bundle of vegetables. * *p <* 0.10, ** *p <* 0.05, *** *p <* 0.01

### 3.3. Bid heterogeneity

The study further investigated the influence of varying scenarios on consumers’ willingness to pay (WTP) premiums for convenience and safety when purchasing vegetables. The research specifically examined premiums for: (1) enhanced hygiene (whole vegetables in improved vs. traditional market stalls); (2) convenience within an improved stall (plucked vs. whole vegetables); and (3) convenience within a traditional stall (plucked vs. whole vegetables). The analysis further considered the impact of demographic and socio-economic factors, including marital status, gender, presence of children under five, household income, and employing a domestic helper.

The results show significant variations in WTP across demographic and socio-economic variables depending on the scenario (Table 5). For the safety premium (whole vegetables in improved stalls), shoppers who employed domestic help and had a higher income submitted a lower bid than those without domestic help and with a lower income (p < 0.01). Also, women submitted higher bids than men for safe vegetables (p < 0.01). Regarding the convenience combined with safety attributes, it was observed that high-income and married shoppers offered lower bids than low-income and single shoppers (p < 0.05). Finally, for the convenience in traditional stalls, women offered higher bids (p < 0.01), married shoppers offered lower bids (p < 0.01), and high-income shoppers offered lower bids (p < 0.01).

**Table 5:** Differences in mean bid prices based on socioeconomic and demographic factors, t-test, n = 417.

Type of premium	Has a domestic helper	Gender (female)	Marital status (married)	Has children below 5 years	High income
Premium for safety (whole vegetable in improved vs traditional stall)	−1.82^***^ (0.65)	1.97^***^ (0.66)	−0.75 (0.51)	−0.27 (0.50)	−2.13^***^ (0.54)
Premium for convenience in an improved stall (whole vs plucked vegetable)	0.77 (0.73)	2.37^***^ (0.74)	−3.63^***^ (0.56)	−1.13^**^ (0.56)	−2.05^***^ (0.61)
Premium for convenience in a traditional stall (whole vs plucked vegetable)	−0.34 (0.60)	2.03^***^ (0.61)	−1.62^***^ (0.47)	0.10 (0.46)	−3.41^***^ (0.50)

Notes: Standard errors in parentheses. ^*^ p < 0.10, ^**^ p < 0.05, ^***^ p < 0.01

### 3.4. Factors influencing willingness to pay

The Tobit model analysis identified several factors influencing consumers’ WTP for whole vegetables sold in an improved stall (hygiene attribute; Table 6). Being female (p < 0.05), older age (p < 0.01), and having a vegetarian household member (p < 0.01) were negatively associated with the maximum price respondents were willing to pay for whole vegetables in an improved stall. However, married shoppers (p < 0.05) and shoppers with a domestic helper (p < 0.01) were willing to pay more for the hygiene attribute, as signified by using whole vegetables in an improved stall.

**Table 6:** Tobit model estimates for factors influencing the purchase of plucked and whole vegetables in an improved stall.

Variable	Whole			Plucked		
	Coeff.	*p*-value		Coeff.	*p-*value	
Female respondent (yes = 1)	−1.97	0.024	**	−2.98	0.008	***
Respondent age (>35 years = 1)	−3.17	<0.001	***	−3.88	<0.001	***
Post-secondary education (yes = 1)	−0.12	0.741		1.46	0.002	***
Married (yes = 1)	1.52	0.030	**	6.33	<0.001	***
Household size (number)	0.11	0.323		−0.15	0.365	
Household has a vegetarian member (yes = 1)	−4.32	<0.001	***	−0.94	0.285	
Household has domestic helper (yes = 1)	4.62	<0.001	***	2.04	0.075	*
Household has children under 5 (yes = 1)	−1.08	0.117		−0.02	0.980	
Can read English (yes = 1)	2.467	0.071	*	2.97	0.091	*
Can read Swahili (yes = 1)	2.2	0.217		4.25	0.063	*
High income level (yes = 1)	0.73	0.316		3.25	0.001	***
Market outlet frequently visited(1 = formal)	−4.23	<0.001	***	−2.75	0.014	**
Bought plucked vegetables (yes = 1)	1.42	0.074	*	3.21	0.002	***
Concerned about eating contaminated vegetables (yes = 1)	1.08	0.048	**	−0.19	0.783	
Food safety knowledge (index ranging from – 3.8 to 1.2)	−0.23	0.478		−1.00	0.017	**
Product quality attributes (index ranging from – 4.1 to 2.5)	−0.32	0.239		0.18	0.610	
Market attributes (index ranging from −2.5 to 2.7)	−0.72	0.014	**	−0.96	0.010	**
Health perception (index ranging from −9.5 to 0.5)	−0.13	0.592		−0.09	0.776	
Constant	63.97	<0.001	***	56.55	<0.001	***

Note: *** p < 0.01, ** p < 0.05, * p < 0.10

For plucked vegetables sold in improved stalls, the Tobit model results revealed additional significant determinants (Table 6). These included the respondent’s education (p < 0.01) and having a high income (p < 0.01), which were positively correlated with WTP, while food safety knowledge (p < 0.05) exhibited a negative correlation.

## 4. Discussion

### 4.1. Willingness to pay for food safety and convenience

The study shows that shoppers were willing to pay higher prices for both safety and convenience, as reflected in substantially higher bids for upgraded stalls (about 25% price premium) and slightly higher bids for minimally processed vegetables in upgraded stalls (about 5% price premium). These results align with those of previous studies in different African cities, highlighting a growing consumer preference for vegetables sold in modern, hygienic retail environments due to perceived safety and quality. A previous study for Nairobi showed that shoppers were willing to pay more for kale that was perceived as safer, demonstrating the importance of food safety in purchasing decisions in an urban setting [39]. Similarly, research on ALVs in Eldoret, Kenya, revealed that factors like appearance, freshness, and perceived nutritional value influenced willingness to pay, reflecting consumers’ preference for quality [40].

Discussions with vendors revealed that, on average, the daily vegetable sales for whole and plucked products are about USD 7.9 (KES 1000) for peri-urban vendors and USD 15.3 (KES 2000) for urban vendors. Our findings suggest that if a vendor were to adopt improved food safety practices, they could earn an additional USD 0.4 for whole vegetables per day in peri-urban areas and USD 0.9 in urban areas, based on existing sales volumes. Peri-urban and urban vendors could increase their daily sales by USD 0.8 and USD 1.6 per day, respectively, by providing plucked vegetables. This translates into an estimated monthly income of USD 439.4 (up from USD 397.8, i.e., USD 15.3*26 days), for urban vendors selling plucked vegetables in improved stalls, assuming 26 business days per month, as vendors do not work on Sundays. There is also potential for sales volumes to increase as consumers prefer vendors offering convenience and improved hygiene.

Previous studies have shown that the preparation time of ALVs is a significant barrier to their consumption in Kenya [3] and South Africa [41]. The laborious process of plucking and washing several times to remove soil is a deterrent to ALV consumption, reducing market demand in favor of vegetables like tomatoes or eggplants that are easier to clean [2]. Typically, the retail prices of whole and plucked vegetables are similar because plucking is viewed as an after-sales service and a means of building goodwill with customers. This explains why the price premium for convenience is only about 4% on average across stall types. Some shoppers also noted that plucking reduces the weight, as stalks are discarded after plucking, even though they are edible as well. Still, the results confirm that shoppers place a value on convenience. This finding contrasts with a study in urban Uganda, which showed that shoppers there did not prefer packaged vegetables from vendors, despite the time-consuming nature of preparing vegetables [42].

### 4.2. Socioeconomic determinants of willingness to pay for food safety and convenience

Socioeconomic characteristics clearly influenced purchasing decisions. Respondents with post-secondary education and those living in high-income areas were likely to pay more for plucked vegetables in improved stalls (p < 0.01), implying that these groups may have greater awareness of or place greater value on food safety and convenience, or have greater financial means to act on these priorities than those with lower formal education or income. There is an increased awareness of the benefits of ALVs among urban residents in Kenya [43], which could influence food purchasing decisions. Higher-income households tend to allocate more resources to healthier food options [44].

Female and older participants were less willing to pay a premium for whole or plucked vegetables in improved stalls. A previous study highlighted the traditional knowledge and experience of women and elderly people in Kenya in preparing ALVs [45]. It is possible that existing knowledge and skills to mitigate food safety risks, through safe handling, hygienic practices in the home, and cooking methods, may equate to a lower emphasis being placed on market stall hygiene. Women are generally more aware of health risks and food safety practices of vendors [46]. As such, they may be skeptical of vendor practices, particularly in urban food environments.

### 4.3. Implications for development

The study found that market infrastructure and hygiene practices influence consumers’ WTP. These findings reinforce those of a previous study [46] that analyzed food purchasing and consumption behavior among men and women in Ghana, Guinea, India, Kenya, and Tanzania. They reported that the physical environment, including retail conditions, vendor appearance, food hygiene practices, and environmental sanitation, helped in signaling vendors’ adherence to food safety regulations.

These results point to the importance of infrastructure upgrades and increasing the capacity of vendors and other value chain actors in food hygiene. The cost of the improved stall type examined in this study was $278, but this could be much lower if produced at scale. However, individual vendors cannot improve aspects such as the floor or the roof of the market, or the sanitary facilities. Such improvements will require external support. Since most retail markets are owned and managed by local governments, they need to invest in these areas. The study’s findings suggest that some of the investment costs could be recovered from market vendors through higher stall fees, which would be passed on to customers. Doing so would increase vegetable prices in the market, potentially impacting low-income households who already spend most of their disposable income on food. Therefore, there is a strong case for public investment in market upgrades, as it would benefit the population overall.

### 4.4. Study limitations

This study assumed that improved stall conditions can enhance food safety and reduce microbial contamination in fresh vegetables. This is not necessarily the case, as microbials may enter the supply chain at the field or farm level. The study did not test microbial loads in improved and traditional stall types, which is a limitation. The study assesses WTP based on perceived safety—as signaled by visible hygiene cues such as clean and protective clothing, elevated storage, and handwashing stations—, not on scientifically verified safety improvements. The study also did not consider the effects of improved stall types on product quality attributes other than safety. Despite this limitation, the study contributes to understanding how stall hygiene shapes consumers’ purchasing decisions.

## 5. Conclusion

The study underscores the critical role of stall hygiene, food safety, convenience, and socio-economic factors in shaping urban Kenyan consumers’ willingness to pay for African leafy vegetables in fresh markets. The preference for improved stalls over traditional ones highlights the importance of improved infrastructure and hygiene in consumer decision-making. The Tobit model analysis reveals that consumers’ willingness to pay for whole and plucked vegetables sold in improved stalls is influenced by key factors such as respondents’ gender, age, income, and marital status, highlighting the role of personal characteristics in purchasing decisions. Authorities should prioritize the modernization of market stalls by ensuring proper sanitation facilities, clean water sources, and well-maintained food environments to enhance consumer confidence in vegetables.

## Supporting information

S1 Fig Improved stall (left) and traditional stall (right) in Jubilee market in Kisumu, Kenya; showing contrasts in stall structure and materials, display of produce, and access to water. (DOCX)

S1 Table Food safety knowledge score. (DOCX)

S2 Table Food safety perception score. (DOCX)

S3 Table Product quality and socio-economic attributes. (DOCX)

S1 Checklist Inclusivity-in-global-research-questionnaire. (DOCX)

S1 Data https://dataverse.harvard.edu/dataset.xhtml?persistentId=doi:10.7910/DVN/7Z9ADG.
(DOCX)
