# Supplementary material for: The price of safety and convenience: Urban shoppers’ willingness to pay for hygienic market stalls and minimal processing of leafy vegetables in Kenya
Source: PLoS One. 2026 Mar 10;21(3):e0340495. doi: 10.1371/journal.pone.0340495 (PMC12974836; doi:10.1371/journal.pone.0340495)
Supplement: S1 Fig — (DOCX) [file pone.0340495.s001.docx]

Figure S1: Improved stall (left) and traditional stall (right) in Jubilee market in Kisumu, Kenya; showing contrasts in stall structure and materials, display of produce, and access to water.


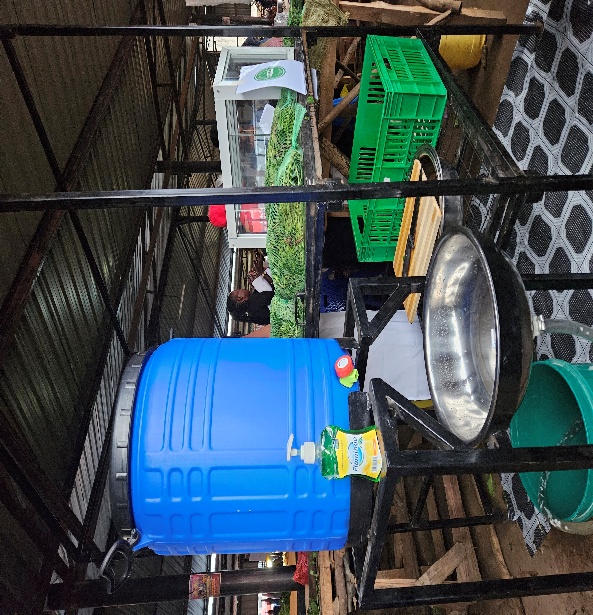

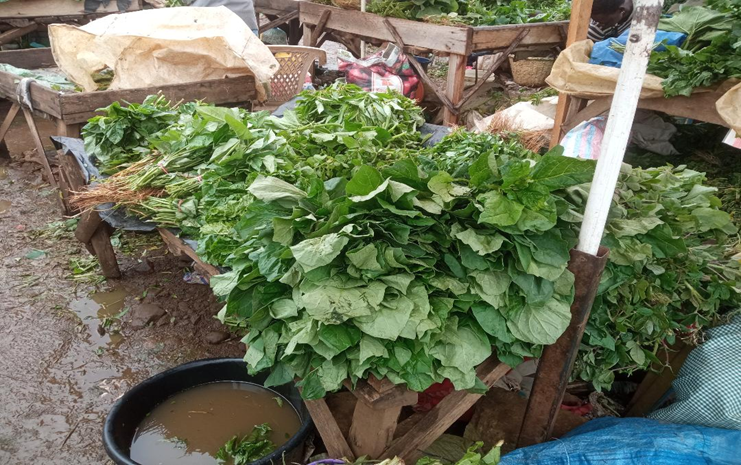


Notes: The total cost of the improved stall was about USD 278.
