# Supplementary material for: The price of safety and convenience: Urban shoppers’ willingness to pay for hygienic market stalls and minimal processing of leafy vegetables in Kenya
Source: PLoS One. 2026 Mar 10;21(3):e0340495. doi: 10.1371/journal.pone.0340495 (PMC12974836; doi:10.1371/journal.pone.0340495)
Supplement: S1 Table — (DOCX) [file pone.0340495.s002.docx]

Table S2: Food safety knowledge score, 1=yes, 0=no, n=417

| **Food Safety Knowledge** |  |  |
| --- | --- | --- |
| Hand hygiene can reduce vegetable contamination (+) | 1.01 (0.11) |  |
| Dust causes vegetable contamination (+) | 1.04 (0.20) |  |
| Consumption of raw/uncleaned vegetables may lead to a foodborne illness that can cause vomiting and diarrhoea (+) | 1.09 (0.30) |  |
| Using clean water to wash vegetables makes vegetables safe (+) | 1.00 (0.07) |  |
| A handler with diseases such as diarrhoea and flu poses a risk of food contamination (+) | 1.05 (0.25) |  |
| Pathogenic bacteria in vegetables are killed off completely if I cook my vegetables (-) | 1.25 (0.52) |  |
| Pathogenic bacteria are only on vegetables from unhygienic stalls (-) | 1.62 (0.50) |  |
| Vegetable contamination from chemicals such as pesticides can cause illnesses (+) | 1.02 (0.15) |  |
| Food from pathogenic bacteria can lead to serious health consequences (+) | 1.01 (0.08) |  |
| Washing cutting boards and knives with a cleaning agent can reduce bacteria (+) | 1.03 (0.17) |  |
| Vegetable contamination from stall cannot cause foodborne illness (-) | 1.87 (0.37) |  |
