# Supplementary material for: The price of safety and convenience: Urban shoppers’ willingness to pay for hygienic market stalls and minimal processing of leafy vegetables in Kenya
Source: PLoS One. 2026 Mar 10;21(3):e0340495. doi: 10.1371/journal.pone.0340495 (PMC12974836; doi:10.1371/journal.pone.0340495)
Supplement: S2 Table — (DOCX) [file pone.0340495.s003.docx]

Table S3: Food safety perception score, 1=strongly disagree, 5=strongly agree, n=417

| **Food Safety Perception** |  |  |
| --- | --- | --- |
| Frequent cleaning of the display tables maintains the safety of vegetables (+) | 4.83 (0.46) |  |
| Frequent cleaning of the tools and equipment maintains the safety of vegetables (+) | 4.82 (0.44) |  |
|  |  |  |
| Placing vegetables on a sack or mat on the ground reduces safety (+) | 4.39 (1.18) |  |
| Using disinfection liquid and soap when cleaning hands maintains the safety of vegetables (+) | 4.65 (0.79) |  |
| Using a stainless-steel tray for displaying and washing vegetables does not affect the safety of vegetables (-) | 2.04 (1.48) |  |
| Wearing a mask and an apron is not important for vegetable sellers (-) | 1.37 (0.78) |  |
| Cleaning hands every time after touching money does not affect the safety of vegetables (-) | 1.93 (1.50) |  |
| Frequent cleaning of retailer’s hands does not affect the safety of vegetables (-) | 1.81 (1.38) |  |
| It is ok to continue cutting vegetables after being cut by a knife (+) | 1.12 (0.47) |  |
