# Supplementary material for: The price of safety and convenience: Urban shoppers’ willingness to pay for hygienic market stalls and minimal processing of leafy vegetables in Kenya
Source: PLoS One. 2026 Mar 10;21(3):e0340495. doi: 10.1371/journal.pone.0340495 (PMC12974836; doi:10.1371/journal.pone.0340495)
Supplement: S3 Table — (DOCX) [file pone.0340495.s004.docx]

Table S4: Product quality and socio-economic attributes, 1= very unimportant, 5=very important, n=417

| **Attribute** | **Score** |  |
| --- | --- | --- |
| **Product quality:** |  |  |
| - Freshness | 4.85 (0.40) |  |
| - Nutritional value | 4.41 (0.99) |  |
| - Colour | 4.71 (0.60) |  |
| - Damage e.g., rot or diseased | 4.31 (1.03) |  |
| - Texture | 4.11 (1.21) |  |
| - Taste | 3.90 (1.44) |  |
| - Odour | 3.85 (1.40) |  |
| - Shape of leaves | 3.29 (1.61) |  |
| - Wholly uprooted/plucked | 3.16 (1.71) |  |
| - Appearance that suggests chemical fertilizer or insecticide was used | 3.14 (1.74) |  |
| - Origin | 2.82 (1.73) |  |
| **Market:** |  |  |
| - Hygiene of the stall | 4.59 (0.88) |  |
| - Size of the bunch | 3.74 (1.63) |  |
| - Location of the vegetable outlet | 3.61 (1.60) |  |
| - Price of vegetables | 3.41 (1.64) |  |
| - Relationship with vendor | 2.93 (1.73) |  |
| - Gender of seller | 1.90 (1.42) |  |
