# Supplementary material for: The price of safety and convenience: Urban shoppers’ willingness to pay for hygienic market stalls and minimal processing of leafy vegetables in Kenya
Source: PLoS One. 2026 Mar 10;21(3):e0340495. doi: 10.1371/journal.pone.0340495 (PMC12974836; doi:10.1371/journal.pone.0340495)
Supplement: S1 Checklist — (DOCX) [file pone.0340495.s006.docx]

The price of safety and convenience: urban shoppers’ willingness to pay for hygienic market stalls and minimal processing of leafy vegetables in Kenya

The dataset for this work is available at the Harvard Dataverse website.

Data S1: https://dataverse.harvard.edu/dataset.xhtml?persistentId=doi:10.7910/DVN/7Z9ADG
